# Highly Sensitive Ultrathin Flexible Thermoplastic Polyurethane/Carbon Black Fibrous Film Strain Sensor with Adjustable Scaffold Networks

**DOI:** 10.1007/s40820-021-00592-9

**Published:** 2021-01-25

**Authors:** Xin Wang, Xianhu Liu, Dirk W. Schubert

**Affiliations:** 1 Institute of Polymer Materials, Friedrich-Alexander-University Erlangen-Nuremberg, Martensstr. 7, 91058 Erlangen, Germany; 2 National Engineering Research Center for Advanced Polymer Processing Technology, Zhengzhou University, Zhengzhou, 450002 People’s Republic of China; 3 Bavarian Polymer Institute, Dr. Mack-Strasse 77, 90762 Fürth, Germany

**Keywords:** Strain sensor, Electrospinning, Electronic skin, Fitting model

## Abstract

In recently years, high-performance wearable strain sensors have attracted great attention in academic and industrial. Herein, a conductive polymer composite of electrospun thermoplastic polyurethane (TPU) fibrous film matrix-embedded carbon black (CB) particles with adjustable scaffold network was fabricated for high-sensitive strain sensor. This work indicated the influence of stereoscopic scaffold network structure built under various rotating speeds of collection device in electrospinning process on the electrical response of TPU/CB strain sensor. This structure makes the sensor exhibit combined characters of high sensitivity under stretching strain (gauge factor of 8962.7 at 155% strain), fast response time (60 ms), outstanding stability and durability (> 10,000 cycles) and a widely workable stretching range (0–160%). This high-performance, wearable, flexible strain sensor has a broad vision of application such as intelligent terminals, electrical skins, voice measurement and human motion monitoring. Moreover, a theoretical approach was used to analyze mechanical property and a model based on tunneling theory was modified to describe the relative change of resistance upon the applied strain. Meanwhile, two equations based from this model were first proposed and offered an effective but simple approach to analyze the change of number of conductive paths and distance of adjacent conductive particles.

**Supplementary information:**

The online version of this article (10.1007/s40820-021-00592-9) contains supplementary material, which is available to authorized users.

## Highlights

- The sensors displayed high sensitivity (8962.7), fast response time (60 ms), outstanding stability and durability (> 10,000 cycles) and widely workable stretching range (0–160%).
- A theoretical approach was used to analyze mechanical property, and a model based on tunneling theory was modified to describe the relative change of resistance.
- Two equations were proposed and offered an effective but simple approach to analyze the change of number of conductive paths and distance of adjacent conductive particles.

**Supplementary information:**

The online version of this article (10.1007/s40820-021-00592-9) contains supplementary material, which is available to authorized users.

## Graphical abstract

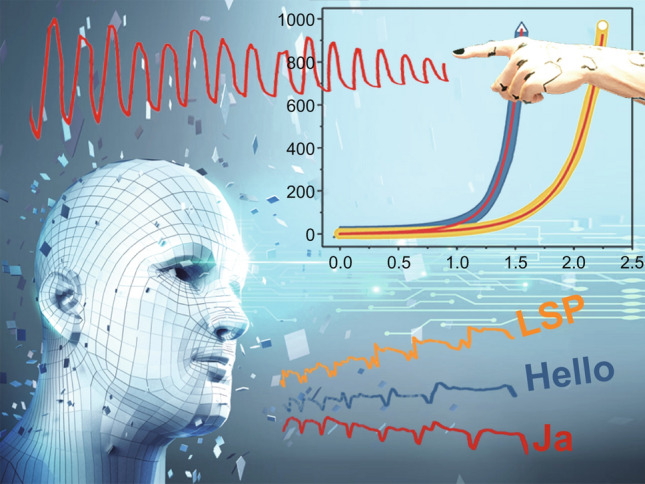

## Introduction

Over the last decades, strain sensors have attracted considerable attention in both academia and industry. With the popularity of various intelligent electronic devices, strain sensors have been widely exploited as flexible electronic skin, human activities monitoring, speech recognition and intelligent robotics [1–8]. For these applications, resistive-type strain sensors based on conductive polymer composites (CPCs) are required in terms of direct signal acquisition system, high sensitivity and low fabrication cost process. Comparing with conventional metals and semiconductors sensor, flexible polymer substrate sensors show excellent stretchability (strain ≥ 50%) and sufficiently flexible to satisfy the complicated and repetitive wide range motions. Therefore, it is preferable to choose different flexible polymer materials as matrix, such as polydimethylsiloxane (PDMS) [9–11], Ecoflex [12] and thermoplastic polyurethane (TPU) [13, 14].

In recent years, the researchers have been extensive support about the production of high-sensitive strain sensors with low cost and simplified processing. Amjadi et al. [15] have fabricated a PDMS elastomer strain sensor with layer-by-layer sandwich. However, the limited detection range (strain = 70%) of this sensor cannot meet the standard of requirements of the human motions. Wang et al. [16] published a PU yarn sensor with coating 8 times single-wall carbon nanotubes (SWCNTs) suspensions. Wei et al. [17] introduced wrinkled microstructure to fabricate a strain sensor with wide detection range and good durability. Unfortunately, complex preparation processes and high cost make limitations of widely application in intelligence terminal devices.

A high-performance strain sensor meeting for requirement of intelligence terminals should present deformation of conductive networks under stress stimuli obviously. Meanwhile, sensors need to keep structural integrity maintain the efficient conductive paths upon large stretching strain and multi-loading cycles. In addition, there is no doubt that simplified processing and lower cost are important as well [5, 18]. Electrospinning has been considered as a versatile processing method to fabricate polymorphic fibrous production, medical equipment field, filtrations and flexible electronic devices due to its simple, stable, continuous and efficient process character [19, 20]. Based on specific spinning process with high voltage, a special collection device is necessary coupling with electrospinning machine for various productions. The stress field provided by collection device is an important factor on interweave and overlap of electrospinning fibers. For polymer material processing, the relationship among stress field, structure of polymer and production properties is close and worth research [21–23]. However, for electrospinning flexible strain sensors, the effect of processing conditions of the collection device on strain sensor sensitive performance has few reported.

In this work, an electrospinning TPU fibrous film was chosen as the polymer matrix to produce a strain sensor. For conductive carbon nano-fillers of CPCs, they are widely used that zero-dimension carbon black (CB) [24, 25], one-dimensional carbon nanotubes (CNTs) [26–29] and carbon fibers (CFs) [30], and two-dimensional graphene and MXene [31–34]. Because of the massive industrial production, processing convenience and economic availability, conductive CB particles were used. Herein, two types of electrospinning TPU fibrous film were fabricated by controlling rotating speed of collection device in 100 and 200 rpm (named TPU-100 or TPU-200 samples) to enlarge the stretching range. The conductive paths based on CB particles were introduced in TPU fibrous film through ultrasonication method to improve sensing stability (named RS-100 or RS-200 samples). The impact of different rotating speed on scaffold network structure and conductive paths forming of TPU/CB strain sensor was studied. To investigate the distinction of electromechanical property, strain sensing tests of RS-100 and RS-200 samples were studied. The results indicated that the TPU/CB strain sensor with 100 rpm is sufficient to meet the requirement for application at large strain. The morphologies of RS-100 and RS-200 strain sensors were observed, indicated various rotating speeds result in the difference of scaffold network structures. The fiber diameter and scaffold interval area of two TPU/CB strain sensors play a great impact of sensitivity during stretching. In addition, the hysteresis of two TPU/CB strain sensors was studied, suggesting the differences in mechanical behavior of materials caused by different structures [35–38]. The dynamic response behaviors of RS-100 strain sensor were explored further to evaluate the repeatability and durability (10,000 cycles). A new mathematic model adapted from the tunneling theory was provided to describe the relative change of resistance ($\Delta R/R_{0}$) upon the strain. Based on parameter calculated from this mathematic model, the variation distance of adjacent conductive particles and the number of conductive paths among the stretching process can be predicted accurately. Furthermore, TPU/CB strain sensors samples were made into simple application to demonstrate human motions monitoring actual, including finger bending, muscle tremor and speaking measuring.

## Experimental Section

### Materials

Carbon Black (CB) was Printex XE2 from Evonik Degussa, with a specific surface area of 900 m^2^/g measured by the BET-method (according to data sheet). The mean diameter of the primary CB particles was around 35 nm and the density at room temperature was 2.13 g cm^−3^. Thermoplastic polyurethane (TPU Elastollan 1180A) with a density of 1.12 g cm^−3^, was provided by BASF Co. Ltd. *N,N*-Dimethylformamide (DMF) was supplied by Merck KGaA, Darmstadt, Germany, and tetrahydrofuran (THF) was bought from Carl Roth GmbH, Co. Ltd. Karlsruhe, Germany. All of the materials were used as received without any purification.

### Preparation of the Ultrathin TPU Fibrous Film

In order to prepare the TPU film, a modified electrospinning apparatus was used comprising of a high voltage supplier (60 kV, Linari Engineering S.r.l., Valpiana, Italy) and a syringe pump (Linari Engineering S.r.l., Valpiana, Italy). First, TPU granules were dissolved in DMF/THF mixed solution with a certain ratio of 10 wt% (the mass ratio of DMF/THF is 1:1). The mixture was mechanically stirred for 3 h to obtain a homogeneous spinning solution at room temperature. Second, a 10 mL capacity syringe with an 18G needle was filled by the resulting TPU solution for electrospinning. For the production of TPU fibrous film, a grounded roller coated by a layer of aluminum foil was used as the collection device with applied rotating speed, a direct current positive voltage of 25 kV was applied between the pinpoint and the collection device. The feed rate of the TPU spin dope was set as 6 mL h^−1^, the feed time was 90 min and the relative humidity was 40%. Here, TPU fibrous film was fabricated under rotating speed of 100 and 200 rpm. In this work, as shown in supporting information Figs. S1 and S2, it is against to fabricate a strain sensor with uniform microstructure and good electrical conductive that low level applied rotating speed of collection device (≤ 50 rpm). The distance between the spin syringe and the collection device was 20 cm. After electrospinning, TPU fibrous film with thickness about 50 μm was prepared (shown in Fig. S3).

### Fabrication of the Ultrathin TPU/CB Strain Sensors

Then CB particles were conducted onto TPU fibrous film through ultrasonication process, which is an economical fabrication method for the flexible strain sensors. First, required quantities CB was dispersed in deionized water (5 mg mL^−1^) by mechanical stirring treatment for 1 h at room temperature. Second, for ease of operation, the TPU film was cut into segments with 60 × 10 mm^2^. Third, the TPU film was immersed into dispersed CB suspension and treated by ultrasonication for 3 h. Finally, the TPU/CB fibrous film was washed for three times by deionized water, and then dried at 80 °C for 1 h in an oven. For convenience of testing, TPU fibrous film with rotating speed of 100 and 200 rpm was labeled as TPU-100 and TPU-200. TPU/CB strain sensors fabricated under 100 and 200 rpm were named as RS-100 and RS-200. To ensure the accuracy of the results, more than 6 specimens in each experiment were tested.

### Characterization

The surface morphology of the samples was subsequently analyzed using a scanning electron microscopy (SEM, Zeiss Compact) with a secondary electron detector at an accelerating voltage of 2 kV. Tensile testing was evaluated on a universal test machine controlled by a custom-built computer with a tensile speed of 5 mm/min. The electromechanical behavior of the TPU/CB strain sensor was recorded on the universal test machine (Zwick Z050) coupled with a Keithley 6487 Pico ammeter at a constant voltage 1 V digit at room temperature, simultaneously. The actual CB volume fraction of TPU/CB strain sensor was measured by utilizing a thermogravimetric analysis (TGA Q5000, TA instruments) under a nitrogen atmosphere, with a heating rate 10 °C min^−1^ from 30 to 600 °C. An isolate clamper of the tensile testing machine was used to fasten TPU/CB sensors samples, as the applied length is 30 mm. For more accretive experimental results, both ends of the strain sensor samples were attached copper tape as electrode, and covered by insulated sheets. To obtain the *I–V* curves of the specimens, static resistance measurement was investigated through Pico ammeter individual, the applied voltage varies from − 5 to + 5 V. Long-term stability test of TPU/CB sensor for 10,000 stretching–releasing cycles toward the strain variation from 0 to 10% under 100 mm/min tensile speed.

## Results and Discussion

### Fabrication of High-Sensitive Ultrathin Flexible TPU/CB Strain Sensors

**Fig. 1:**
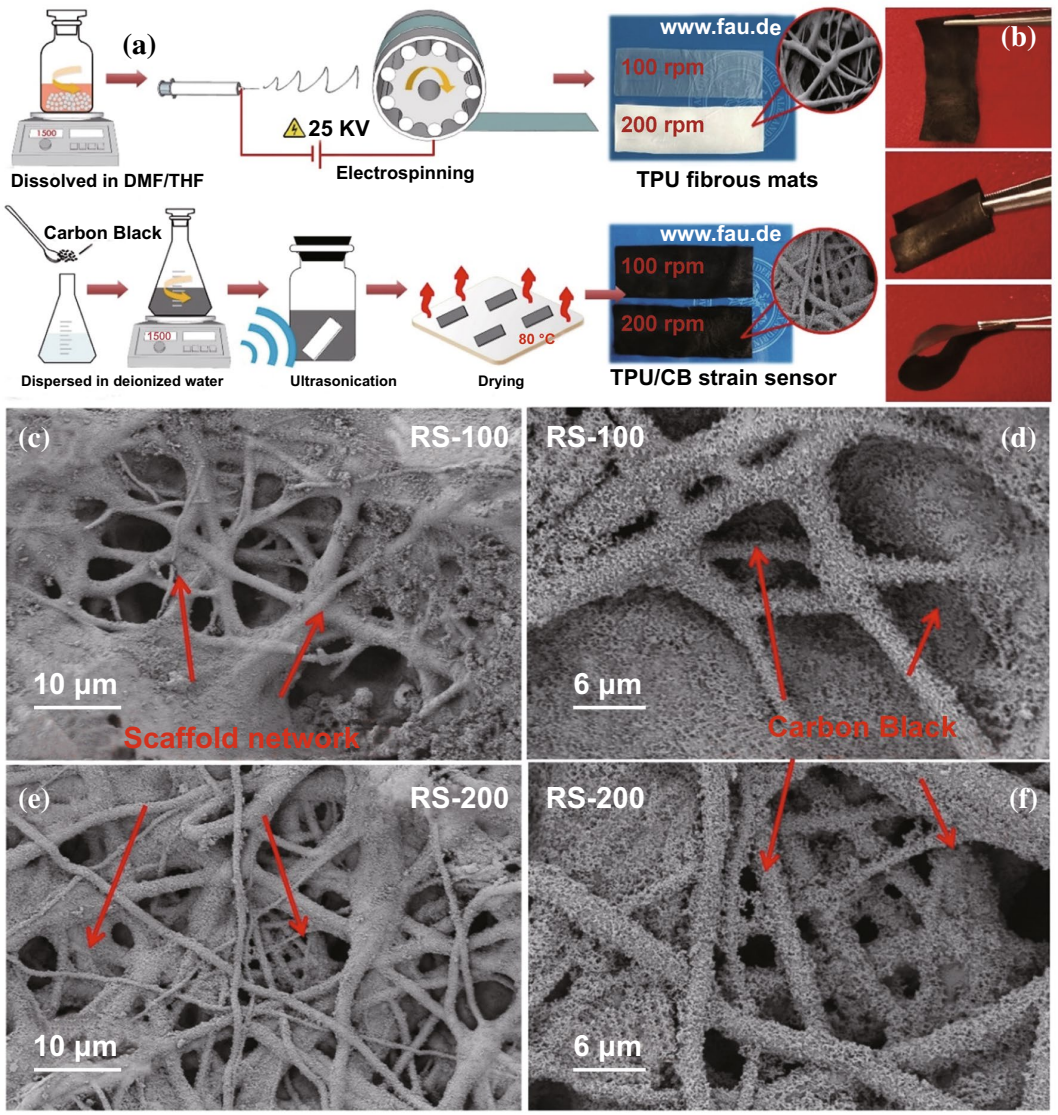
Fabrication and characterization of the TPU/CB strain sensor. **a** Schematic diagram of production process and **b** flexibility of TPU/CB strain sensor. SEM images of TPU/CB strain sensor with **c-d** 100 rpm and **e**–**f** 200 rpm. (Color figure online)

The fabrication process of a highly stretchable, sensitive and wearable TPU/CB strain sensor is shown in Fig. 1a. First, TPU fibrous films were prepared by electrospinning with various rotating speeds of a particular collection device. Second, CB conductive particles were embedded in TPU fibrous film through a simple ultrasonication treatment. The ultrathin TPU/CB strain sensors present excellent flexibility with various curly shapes (Fig. 1b). Figure 1c, d displays the SEM characterization of the pure TPU film under different rotating speeds. It is found that a scaffold network structure was built by TPU fibers. Additionally, CB particles distributed on the fibers uniformly and firmly for both RS-100 (Fig. 1e) and RS-200 (Fig. 1f) samples. No significant orientation of TPU fibers can be observed whatever RS-100 or RS-200 samples. Interestingly, the TPU-100 fibrous film is evenly and transparent while the color of TPU-200 film is deeper and present white, which should be related to the difference of scaffold network structure of TPU-100 and TPU-200 samples.

### Tensile Strength and Electrical Resistance of TPU/CB Strain Sensors

**Fig. 2:**
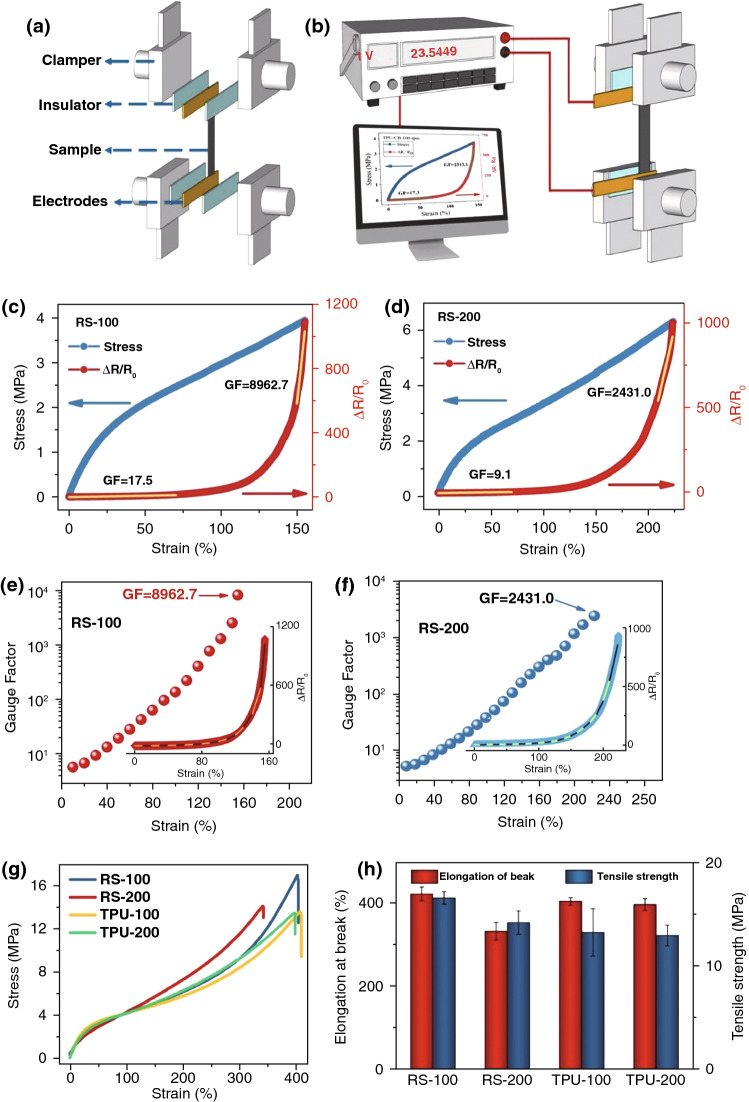
Schematics of **a** the tensile test clamper with a sample and **b** the tensile testing machine coupled with a Pico ammeter. **c**,** d** The normalized change in tensile stress and electrical resistance ($\Delta R/R_{0}$) vs. strain as well as **e**, **f** GF for **c**, **e** RS-100 and **d**, **f** RS-200 samples. **g** Typical stress–strain curves and **h** tensile strength and elongation at break of RS-100, RS-200, TPU-100 and TPU-200 samples. Inset: GF of every segment divided by a 10% strain

For CPCs, polymer matrix provides the carriers for conductive particles. Thus, the conductive macro-pathways are built because of the construction and reconstruction of polymer matrix networks. In this paper, sensing–strain performances are investigated toward the applied stress stimulus. The sensitivity of TPU/CB strain sensors was evaluated by the normalized change in electrical resistance $\Delta R/R_{0}$ (where $\Delta R = R - R_{0}$, $R$ is the resistance after deformation and $R_{0}$ represents the original resistance of the samples). To calculate the sensitivity of the TPU/CB strain sensors, a tensile testing machine was coupled with a Pico ammeter (Fig. 2a, b). For the sensing–strain behavior, the stress–strain and sensing–strain curves of different rotating speed TPU/CB strain sensors were plotted with respect to an applied tensile strain. As shown in Fig. 2c, d, for the stress–strain behaviors, the stresses of RS-100 and RS-200 strain sensors improved gradually with the increasing strain. It is noteworthy that the stress values are 3.79 and 4.77 MPa under 150% strain for the RS-100 and RS-200 strain sensors, respectively. Moreover, the largest tensile strains were 155% for RS-100 and 225% for RS-200 as electrical signals can be observed stably, which suggesting that RS-200 sensors have better mechanical performance. For the sensing–strain behaviors, the $\Delta R/R_{0}$ values of the RS-100 and RS-200 samples present significant differences. The $\Delta R/R_{0}$ values of the RS-100 and RS-200 samples both increased exponentially with the increasing strain. This is attributed to the partial breakdown of electrical conductive pathways and the gradual increase in the separation between CB, based on the model of the tunneling mechanism, both leading to an increase in resistance. Obviously, in comparison with the RS-200 fibrous sensor, the RS-100 fibrous sensor exhibits outstanding responsivity. It can be ascribed to the differences of scaffold network microstructures and the various distribution densities of the conductive particles, which lead to a great variation of the conductive networks in the process of dynamic tension. Generally, the sensitivity of stretchable strain sensors is evaluated by gauge factor (GF), defined as the ratio of the relative change in resistance to mechanical strain [6].GF is analyzed by the slope of the variation curve, which can be calculated by Eq. (1):

1 $${\text{GF }} = \frac{{{\text{d}}\left( {\Delta R/R_{0 } } \right)}}{{{\text{d}}x}},$$

where $\Delta R/R_{0}$ and *x* represent the normalized change in electrical resistance and mechanical strain, respectively.

To compare the sensitivity of RS-100 and RS-200 strain sensor samples, a linear increase in ΔR/R_0_ was defined between 0 and 70% strain, corresponding to a GF of 17.5 for RS-100 and 9.1 for RS-200 (Fig. 2c, d). After a transition legion, RS-100 and RS-200 samples reached their maximum stretch. The high deformation will lead to a violent change with extremely high GF of 8962.7 for RS-100 at the largest strain for 155%, while the GF for RS-200 is not significant (GF = 2431.0 at strain for 225%). As shown in Fig. 2e, f, to compare the conductive sensitivity of two samples clearly and accurately, every segment GF of RS-100 and RS-200 samples divided by a 10% strain were calculated by linear fitting from electrical resistance as a function of strain. It is obvious that GF of RS-100 strain sensor is much higher than that of RS-200 under the same strain segments. For example, the GF values of the RS-100 and RS-200 samples were about 186.6 and 43.5 at a strain of 100%, respectively. It is obviously that the sensitivities stay related with the mechanical properties of stretchable strain sensors. The deformation of the microstructures is crucial for fine-tuning sensing–strain performance because of the mismatch of the mechanical properties of matrix and conductive particles [3, 39]. To investigate the effect of rotating speed in processing on mechanical properties of TPU/CB strain sensors, a series of monotonic tensile tests were performed. As shown in Fig. 2g, h, the typical stress–strain curves and histograms of mechanical properties were depicted. Overall, stress–strain curves of all samples are similar, specific strength and elongation at break of the four samples have no significant change. This indicates that TPU fibrous film is not damaged under ultrasonic treatment and impact of CB. In Fig. 2g, it is worth mentioning that the tensile strength curve of TPU-200 sample (green line) is higher than tensile strength curve of TPU-100 sample (yellow line) during 100–400% strain in stretching processing while the strengths of them are similar under lower deformation (< 100% stain). In addition, as shown in Fig. 2h, the tensile strengths for TPU-100 and TPU-200 samples are similar (~ 13 MPa) while the elongation at break of TPU-100 is almost 11% higher than that of TPU-200. It means that the impedance capability of TPU-200 sample is stronger than that of TPU-100 sample at the case of a larger stretching, while there is not much difference in its own tensile strength because of the limitation of its original pure material mechanical properties. The tensile strengths are observed to improve significantly after the introduction of CB particles as compared with those of pure TPU. The large improvement of mechanical properties is attributed to the homogeneous dispersion of CB in the TPU matrix, and the hydrogen bonding between CB and TPU fibrous film. In addition, as shown in Fig. 1d, e, CB particles were distributed evenly through the TPU matrix without obvious aggregation and restacking. It is known that aggregation of conductive fillers is a key factor of mechanical properties and electrical conductivity reducing significantly. The elongation at break of RS-100 is 422% ± 16%, which is higher than that of RS-200 (331% ± 21%) obviously. The tensile strength of RS-100 (16.5 ± 0.6 MPa) is larger than that of RS-200 (14.1 ± 1.1 MPa) as well, which can be ascribed from the benefits of different stereoscopic scaffold network microstructures of TPU fibrous networks.

### Morphology of TPU/CB Strain Sensors

For CPCs, the different microstructures play a crucial role in determining the mechanical properties. Meanwhile, the distinguished mechanical properties are beneficial for the application [2, 5].To investigate the microstructure clearly, the fiber diameters and scaffold interval areas of TPU/CB strain sensors were measured by using a software ImageJ and the histograms were fitted by normal distribution.

**Fig. 3:**
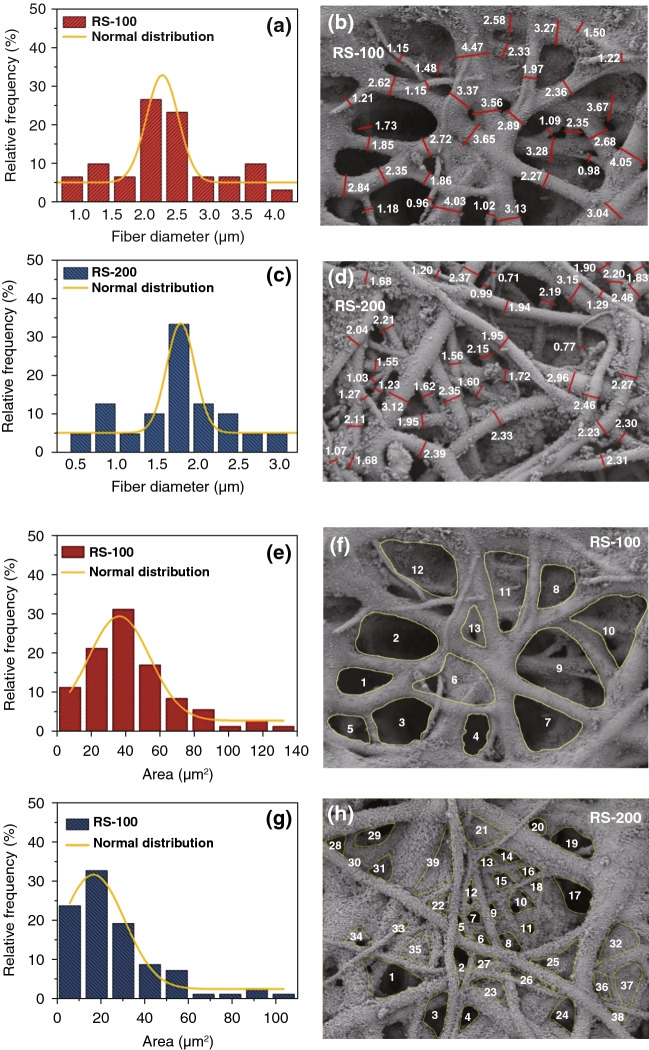
Study of the distributions of fiber diameters and scaffold network interval areas of TPU/CB strain sensor. The histograms of **a**, **c** fiber diameter distribution and **e**, **g** scaffold interval areas of **a**, **e** RS-100 and **c**, **h** RS-200 samples as well as the corresponding SEM images of **b**, **f** RS-100 and **d**, **h** RS-200 samples. The red lines and white numbers are diameters and actual lengths (µm), respectively, and the scaffold interval areas were labeled and signed in yellow

Figure 3 shows the distributions of fiber diameters and scaffold interval areas on the RS-100 and RS-200 strain sensors. As shown in Fig. 3a–d, with the rotating speed increasing from 100 to 200 rpm, the corresponding diameters of TPU fiber decreases obviously. It can be known from the histograms of normal distribution fitting (Fig. 3a, c). Fiber diameters of RS-100 are mostly distributed in an interval value of 2.28 ± 0.04 µm, while the distribution interval of RS-200 sample is more concentrated at 1.77 ± 0.05 µm. In Fig. 3e, f, the areas of scaffold network structure interval are calculated and counted. At the same magnification, the number of networks of RS-100 sample is lower than the total number of them in RS-200 sample. While the interval areas of RS-100 sample are larger than them of RS-200 sample (Fig. 3f, h). As shown in normal contribution of areas of scaffold internal in Fig. 3e, g, the value of RS-100 is 36.58 ± 1.43 µm^2^. While the normal contribution value of RS-200 is 16.91 ± 1.33 µm^2^, almost the half of the value of RS-100. For TPU/CB strain sensors, TPU fibrous film is weaved by staggered arrangement TPU fibers with CB particles dispersing homogeneous on, which constitutes a three-dimensional (3D) conductive network structure with stereoscopic internal spaces. For electrospinning process, a stress field was formed by the high-speed rotating collection device, TPU fibers were stretched and rearranged in stress field as well. Generally speaking, the influence of different stress fields on the material processing would be positively reflected in the microstructure of the product [21, 40]. The differences of TPU fiber diameters and interweave-overlap structures based on stress fields make the scaffold network structures of TPU matrix different, leading to conductive paths built by carried CB particles have a significant differentiation during stretching, which present different response sensitivity in the macroscopic. Under the lower rotating speed, 100 rpm, the thicker TPU fibers of 2.28 µm were obtained to build fibrous film with larger scaffold interval structure. When a higher rotating speed applied, 200 rpm, a stronger stress field makes the TPU fibers stretched, leading to the diameters of TPU fibers reduced to 1.77 µm. In addition, within the same feed rate and feed time, the collection device worked more cycles at higher rotating speed, which should fabricate much more layers of TPU fibrous film. It exists that a stress taken to the former layer film from the next layer, which makes the whole TPU fibrous film form a multilayer-networks structure with dense scaffold networks. Comparing with RS-200 strain sensors, RS-100 samples formed a more stereoscopic and distinguishable network structure with larger scaffold interval structure. For pure TPU fibrous film, dense network structure presents a lack of toughness and transmission of light, which indicated the elongation at break of TPU-200 samples is less than TPU-100 samples and the color of TPU-200 samples is deeper than TPU-100 samples. On the other hand, the dense scaffold network structure also has a significant impact on the introduction of CB by ultrasonication method. For RS-100 TPU/CB strain sensors, more typical stereoscopic scaffold network structure and larger scaffold interval made CB particle not only attached on the outmost fibers, but also entered the internal fibers from the gaps of network, which enhanced the strength of CPCs. In the TPU/CB strain sensors, conductive networks formed as CB particles point-point contact. When the CPCs are stretched, the distance between CB particles changes with the superior mechanical flexibility TPU macromolecular chains stretching and wriggling, resulting in the breakage of conductive pathways and the increase in the tunneling distance between CB particles. At the same time, some new conductive pathways are reconstructed. The destruction of the conductive network is predominant in the loading process, so an increasing $\Delta R/R_{0}$ is observed. For RS-100 samples, thicker TPU fibers provide a larger matrix for the combination of CB and TPU. Meanwhile, the three-dimensional and distinguishable network structure results in the tunneling distance closing between CB particles, which means there are more opportunities to construct conductive micro-pathway with TPU fibers as foundation. When the CPCs are stretched, the “bridge framework” of RS-100 samples, stereoscopic scaffold network, is easier to stretch and wriggle, leading to the breakage of conductive pathways, which present quick response in the form of electrical resistance variation. On the contrary, the sensitivity of RS-200 samples is lower because of its dense scaffold network structure and resistance performance to applied stress. Therefore, this work makes it become a great feasibility study that to fabricate a controllable sensitive TPU/CB strain sensor through adjusting the rotating speed of collection device.

### Mechanical Hysteresis Quantification of TPU/CB Strain Sensor

**Fig. 4:**
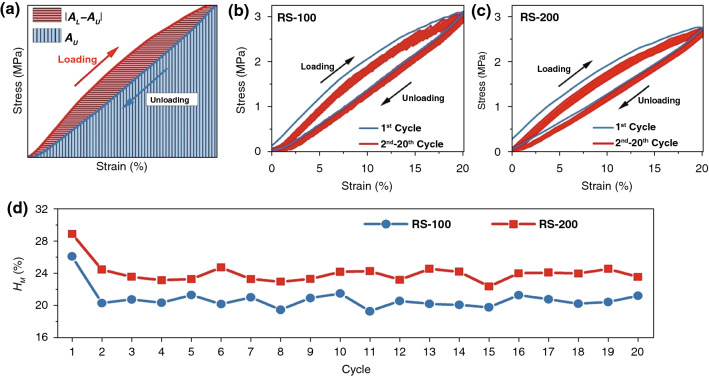
**a** Schematic of mechanical hysteresis ($H_{{\text{M}}}$) quantification; cyclic stress–strain curves of **b** RS-100 and **c** RS-200 stain sensors; **d** mechanical hysteresis ($H_{{\text{M}}}$) of TPU/CB sensors

In actual human movement application and work, it is important to investigate the long-term durability and reliability. Mechanical hysteresis ($H_{{\text{M}}}$) is an effective method which can describe the energy variation of TPU/CB strain sensors because of the formation/ destruction of conductive networks during multi-loading–unloading cycles [35, 36]. In order to summarize the mechanical hysteresis ($H_{{\text{M}}}$), Fig. 4 plots $H_{{\text{M}}}$ for 20 cycles as function of the various applied rotating speeds. $H_{{\text{M}}}$ is quantified for each full cycle comprising the loading and unloading curves, calculated from the stress–strain curves. According to Fig. 4a, the area of ($A_{{\text{L}}}$–$A_{{\text{U}}}$) and the area of $A_{{\text{U}}}$ are represented by red and blue colors, respectively. The value of $H_{{\text{M}}}$ can be computed by the following Eq. (2):

2 $$H_{{\text{M}}} = \frac{{|A_{{\text{L}}} - A_{{\text{U}}} |}}{{A_{{\text{L}}} }},$$

where $H_{{\text{M}}}$ is calculated in a similar fashion by considering the absolute value of the difference in the area under the curve of the loading path ($A_{{\text{L}}}$) and that of the unloading path ($A_{{\text{U}}}$).

Figure 4b, c is the cyclic stress–strain curves of RS-100 and RS-200 strain sensors. The blue curve is the first cycle of stretching and recovery, and the red curves are the second to the twentieth cycles. Figure 4d shows $H_{{\text{M}}}$ for 20 cycles as function of the rotating speed, for TPU/CB strain sensors. It is seen that an increase in the higher rotating speed leads to a decrease in $H_{{\text{M}}}$. At the first cycle, the relatively high $H_{{\text{M}}}$ is ~ 26.1% for RS-100 and ~ 28.9% for RS-200, respectively. In addition, the average $H_{{\text{M}}}$ is reduced to ~ 20.6% for RS-100 and ~ 23.6% for RS-200 in second to twentieth cycles. During the loading process, the scaffold network structure of TPU got stretched and some CB conductive pathways broke. At the same time, it also existed plastic deformation and elastic deformation on TPU under applied stress. During the unloading process, the TPU samples returned to their original states and elastic deformation recovered, while plastic deformation was irreversible due to the hysteresis effect of the composites [35, 36]. After several stretching cycles, the sample reached a balance of hysteresis effect. This can indicate the $H_{{\text{M}}}$ reduced in first cycle and became stable during subsequent cycles regardless of the rotating speed. For RS-100 and RS-200 strain sensors, the reduction of $H_{{\text{M}}}$ for RS-200 is more obvious than $H_{{\text{M}}}$ for RS-100, which means the thicker TPU fibers and specific scaffold network structure make RS-100 samples present less energy consumption and better long-term durability than that in RS-200 samples during multi-recovery process.

### Theoretical Analysis of Conductive Fillers Mechanical Properties and Schematics of Stretching

**Fig. 5:**
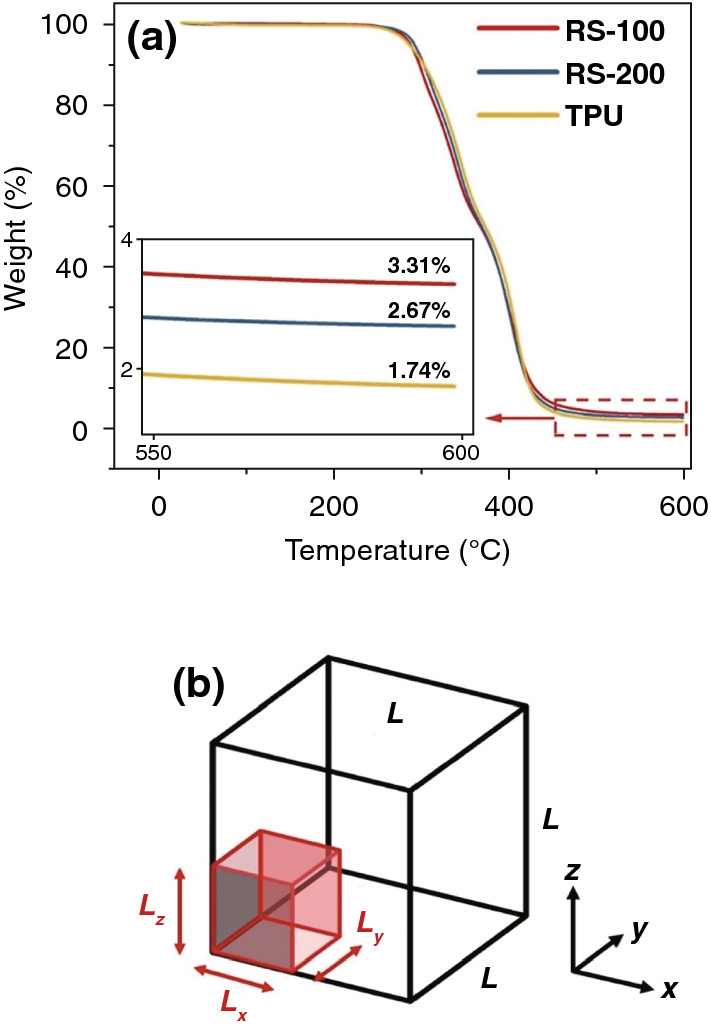
**a** TGA of pure TPU fibrous film and TPU/CB strain sensors. **b** Diagram of the volumes, a ($L \times L \times L$) size filler cube with an additional infinitesimal amount of filler in a smaller ($L_{x} \times L_{y} \times L_{z}$) size cube

In Fig. 5a, the contents of CB for RS-100 and RS-200 samples were measured by the thermogravimetric analysis (TGA), which could examine the content of CB in TPU/CB strain sensor samples. The content of CB anchored on TPU/CB strain sensor ($W_{{{\text{CB}}}}$) can be calculated by the following equations:

3a $$m_{{\text{s}}} = m_{{{\text{CB}}}} + m_{{{\text{TPU}}}}$$

3b $$b \cdot m_{{\text{s}}} = m_{{{\text{CB}}}} + a \cdot m_{{{\text{TPU}}}}$$

4 $$W_{{{\text{CB}}}} = \frac{{m_{{{\text{CB}}}} }}{{m_{{\text{s}}} }} = \frac{b - a}{{1 - a}},$$

where $m_{{{\text{TPU}}}}$ and $m_{{{\text{CB}}}}$ are the weights for pure TPU fibrous film and CB in the strain sensor, respectively. $m_{{\text{s}}}$ is total weight of TPU/CB strain sensor. The remains weight ratios $a$ for pure TPU film and $b$ for TPU/CB strain sensor are measured after thermal degradation shown in Fig. 5a. Thus, the content of CB for RS-100 strain sensor ($W_{1}$) is 1.60 wt% and $W_{2}$ for RS-200 is 0.95 wt% calculated by Eq. (4). The content of CB for RS-100 strain sensor is higher than that of RS-200 strain sensor, which can be ascribed that more CB particles anchored on the thicker TPU fibers and some CB particles entered inside through the larger scaffold interval area.

A very promising theoretical analysis approach to describe mechanical properties of conductive fillers and polymer was published by Schubert recently [41]. In this theoretical approach, as shown in Fig. 5b, a consideration filler cube of size ($L \times L \times L$) contains a low-volume fraction of filler $\Phi$ homogeneously distributed. An additional infinitesimal amount of filler is located in a smaller body of size ($L_{x} \times L_{y} \times L_{z}$). Property $P$ stands for elastic modulus, shear modulus, or viscosity. The equation as follows:

5 $$P\left( \Phi \right) = P_{{{\text{Matrix}}}} \left[ {1 + \left( {\left( {\frac{{P_{{{\text{Filler}}}} }}{{P_{{{\text{Matrix}}}} }}} \right)^{\frac{1}{t}} - 1} \right) \cdot \Phi } \right]^{t} ,\;\;t = \left[ {1 - \frac{{2 \times \frac{L}{{L_{x} }} \cdot \frac{L}{{L_{y} }} \cdot \frac{L}{{L_{z} }} \times \left( {1 - \frac{{L_{z} }}{L}} \right)}}{{\frac{L}{{L_{x} }} \cdot \frac{L}{{L_{y} }} \cdot \frac{L}{{L_{z} }} - 1}}} \right]^{ - 1}.$$

For RS-100 and RS-200 strain sensors, the conductive fillers CB can be regarded as attached to the TPU matrix surface uniformly, which means the fillers just distributed in x–y plane. Therefore, $\frac{{L_{z} }}{L} \to 0$, $\frac{L}{{L_{x} }} \cdot \frac{L}{{L_{y} }} \cdot \frac{L}{{L_{z} }} \to + \infty$, then $t \to - 1$. then substituting ($t \to - 1)$ into Eq. (5),

6 $$P\left( \Phi \right) = P_{{{\text{Matrix}}}} \left[ {1 + \left( {\frac{{P_{{{\text{Matrix}}}} }}{{P_{{{\text{Filler}}}} }} - 1} \right) \cdot \Phi } \right]^{ - 1}$$

after rewriting,

7 $$\frac{1}{P\left( \Phi \right)} = \frac{1 - \Phi }{{P_{{{\text{Matrix}}}} }} + \frac{\Phi }{{P_{{{\text{Filler}}}} }}$$

as Property $P$ stands for elastic modulus, shear modulus, or viscosity, so in this work $P_{{{\text{Filler}}}} > P_{{{\text{Matrix}}}} > 0$. The contents of CB are calculated from Fig. 5a $\left( {W_{1} > W_{2} > 0} \right)$, under the same condition, for the volume fraction of RS-100 ($\Phi_{1}$) and RS-200 ($\Phi_{2}$), $\Phi_{1} > \Phi_{2} > 0$, then

8 $$\frac{1}{{P\left( {\Phi_{1} } \right)}} - \frac{1}{{P\left( {\Phi_{2} } \right)}} = \frac{{\Phi_{1} - \Phi_{2} }}{{P_{{{\text{Filler}}}} }} - \frac{{\Phi_{1} - \Phi_{2} }}{{P_{{{\text{Matrix}}}} }},\;\;\Phi_{1} - \Phi_{2} > 0$$

9a $$\frac{1}{{P\left( {\Phi_{1} } \right)}} - \frac{1}{{P\left( {\Phi_{2} } \right)}} < 0$$

9b $$P\left( {\Phi_{1} } \right) > P\left( {\Phi_{2} } \right) > 0.$$

As shown in Eq. (9), the property $P$ of RS-100 sample is larger than that of RS-200, which stands for elastic modulus, shear modulus, or viscosity. Mathematic proof shows that the benefits of scaffold network structure for RS-100 strain sensor on better tensile property and less energy consumption.

For TPU/CB strain sensor, under applied stress stimulation, the curly TPU fibers are straightened and elongated, which makes the deformation of scaffold network structure, resulted in scaffold interval increasing. The deformations of scaffold network structure and changes of conductive paths for RS-100 and RS-200 strain sensors during stretching are shown in Fig. S6. The red lines represent the conductive paths built by CB particles. For RS-100 strain sensors, original conductive pathways are easier to get breakage than RS-200 samples due to the scaffold network structure with larger scaffold interval. In addition, the dense network structure of RS-200 strain sensor makes it possible that the TPU fibers are entangled to reconstruct a new conductive pathway during stretching. It can be ascribed the conductive paths of RS-100 strain sensors have a more obvious response to deformation.

The foregoing discussion indicated that TPU/CB strain sensors with rotating speeding 100 rpm were more satisfied than RS-200 strain sensors due to the requirement of high sensitivity, wide strain range and long-term durability meeting actual sensing applications. Thus, the related experiments of electromechanical properties were employed by RS-100 strain sensor samples.

### Conductive Sensitivity Performance of TPU/CB Strain Sensor

**Fig. 6:**
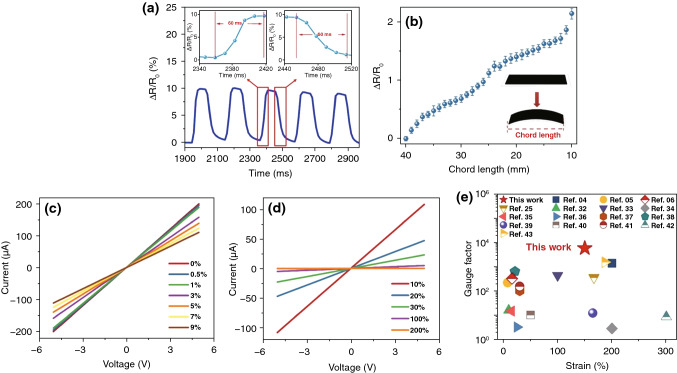
Performance of TPU/CB strain sensor. **a** Time response. Insets: close-up of the selected areas. **b** ΔR/R_0_ as a function of chord length under bending. Inset: the schematic illustration of tension and compression modes. I–V curves at various micro strains (**c)** and large strains (**d)**. **e** Comparison of the GF and maximum strain of TPU/CB strain sensor with those of the counterparts reported in the literature

In Fig. 6a, the TPU/CB strain sensor was loaded with a quasi-transient step strain of 1%. The response time was measured to be short of 60 ms, which was among the highest reported in the literature. In Fig. 6b, the $\Delta R/R_{0}$ of the sensor increased monotonically with the chord length decreasing from 40 to 10 mm by bending samples. This result indicated that the sensor also had favorable ability to detect deformations of bending. The current–voltage (I–V) characteristic of TPU/CB strain sensor with diverse strains is depicted in Fig. 6c, d. The *I–V* curves confirmed strictly to the Ohm's law under both micro strains (from 0 to 9%) and large strains (from 10 to 200%). When the voltage increases gradually from − 5 to 5 V, the linear *I–V* curves confirmed Ohm’s law excellently of TPU/CB strain sensors. This indicated that the significant reliability and widely apply range of TPU/CB strain sensors, which are important to monitor human motion. As mentioned above, the highest GF value can be stabilized at 8962.7 with a 150% tensile strain (Fig. 2e). Figure 6e exhibits the main performance criteria of recently reported typical strain sensors as comparison, indicating the superior performance of TPU/CB strain sensors with the integration sensitivity and wide sensing range, especially for human movement applications [4, 5, 18, 27, 42–53].

### Long-Term Sensing–Strain Properties of TPU/CB Strain Sensor

**Fig. 7:**
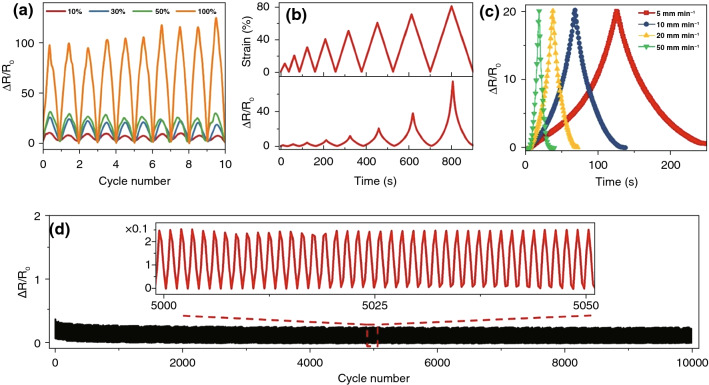
Sensing–strain properties of TPU/CB strain sensor. **a** Multi-cyclic tests respond to repetitive stretching at diverse maximum strains. **b** Strain–time and R/R_0_–time curves during step cyclic deformation with various maximum strains by the same sample. **c** The normalized changes of electrical resistance (${\Delta }R/R_{0}$) at diverse frequencies under 70% strain. **d** 10,000 stretching–releasing cycles toward the strain variation from 0 to 10% under 100 mm/min

To verify stability at high frequencies and fast signal response, dynamic resistance is regarded as a significant feature [45, 51]. As shown in Fig. 7a, electrical cyclic responses under cyclic stretching with various strains (10%, 30%, 50% and 100%) were systematically investigated. The response patterns were identical for different strains, which showed that the TPU/CB strain sensor had an excellent stable and continuous response to the cycling loading. In different cycles, $\Delta R/R_{0}$ almost unchanged under the same load, due to the similar destruction of the conductive networks, indicating the sensitivity and stability of the TPU/CB strain sensors. Figure 7b displays strain–time and $\Delta R/R_{0}$–time curves of TPU/CB strain sensor during stepwise cyclic deformation with maximum strains of 10%, 20%, 30%, 40%, 50%, 60%, 70% and 80% by the same sample. The tendencies of $\Delta R/R_{0}$ are similar with stepwise cyclic strain and $\Delta R/R_{0}$ can return to initial level, which means the recoverability of TPU/CB strain sensors is outstanding. The influence of stretching speed on flexible TPU/CB strain sensor was investigated in detail. As shown in Fig. 7c, it remained stable that the $\Delta R/R_{0}$ of TPU/CB sensor when the test rate increased from 5 to 50 mm min^−1^. As flexible strain sensors, this feature is essential for obtaining a reliable response under different external stimuli. As shown in Fig. 7d, a long-term working life with 10,000 loading/unloading cycles was tested on the electrical response of the TPU/CB strain sensors to demonstrate an extreme stability at the strain of 10% with a strain speed of 100 mm min^−1^. In Fig. 7d, no significant signal fluctuation can be observed, which illustrated excellent reproducibility and durability of the sensors in the practical applications. As mentioned above, TPU/CB strain sensors exhibited high sensitivity, outstanding stretchability, fast response, multi-function and excellent repeatability concurrently.

### Model Fitting for Experiments Results

As proved in previous experiments, the structure of TPU fibrous film gets an obvious affective by rotating speed of collection device for TPU/CB strain sensors. The construction of scaffold network structure takes a greatly influence of entangled TPU macromolecular chains stretching and wriggling. In microstructure level, the total resistance of TPU/CB strain sensor is a function of both the resistance through each conducting particle and the polymer matrix [6, 54]. The number of conducting particles and conducting paths makes an important role between electrodes, which can be shown on $\Delta R/R_{0}$-strain curve intuitively. The total resistance can be described by Eq. (10) [55]:

10 $$R = \frac{{M\left( {R_{{\text{m}}} + R_{{\text{c}}} } \right)}}{N},$$

where $R$, $R_{{\text{m}}}$ and $R_{{\text{c}}}$ are the resistance of composite, the resistance between two adjacent particles and the resistance across one particle, respectively. For conductive polymer composites, as $R_{{\text{m}}}$ is much larger than $R_{{\text{c}}}$, the resistance across the particle can be neglected $\left( {R_{{\text{m}}} + R_{{\text{c}}} \approx R_{{\text{m}}} } \right)$. $M$ is the number of particles forming one conducting path and $N$ is the number of conducting paths, respectively. Based on the tunneling effect model, the composite resistance is described by Eq. (11) [54, 55]:

11 $$R_{m} = \frac{8\pi hs}{{3c^{2} re^{2} }} {\text{exp}}\left( {rs} \right),\;\;r = \frac{4\pi }{h} \sqrt {2m_{{\text{e}}} \varphi }$$

then substituting Eq. (11) into Eq. (10) gives

12 $$R = \frac{M}{N}\left[ {\frac{8\pi hs}{{3c^{2} re^{2} }} {\text{exp}}\left( {rs} \right)} \right],$$

where *h* is the Plank's constant, *c*^2^ the effective cross section, *e* the electron charge, *s* the average distance between adjacent conductive particles. $m_{{\text{e}}}$ and $\varphi$ are the electron mass and the height of tunneling potential barrier, respectively.

As mentioned above, applied stress on the CPCs make the increase in the tunneling distance between CB particles, resulting in the breakage and reconstruction of conductive pathways. Assuming that the tunneling distance varies from *s*_*0*_ to *s* between adjacent particles satisfying a liner function, the number of conductive network changes from *N*_*0*_ to *N* satisfying an exponential function. They can be calculated as follows:

13 $$s = s_{0} \left( {1 + Ax} \right)$$

14 $$N = N_{0} \times {\text{exp}}\left[ {f\left( x \right)} \right],$$

where *s*_*0*_ and *N*_*0*_ are initial particle separation and the number of conductive pathways in initial state, respectively. *x* is applied strain and $f\left( x \right)$ is a function of strain *x*. *A* is a constant which depends on the category of conductive fillers and the measurement condition. Then, $\Delta R/R_{0}$ is obtained by substitution of Eq. (15).

15 $$\begin{aligned} & \frac{\Delta R}{{R_{0} }} = \frac{{R - R_{0} }}{{R_{0} }} = \frac{R}{{R_{0} }} - 1 = \left( {\frac{s}{{s_{0} }} \times \frac{{N_{0} }}{N}} \right)\exp \left[ {r\left( {s - s_{0} } \right)} \right] - 1 \\ & \quad \quad = \left( {1 + Ax} \right)\exp \left[ {r\left( {s - s_{0} } \right) - f\left( x \right)} \right] - 1, \\ \end{aligned}$$

where $\left[ {r\left( {s - s_{0} } \right) - f\left( x \right)} \right]$ can be indicated as the relationship between height of tunneling potential barrier ($\varphi$) and the variations of conductive paths (*N*). set

16 $$wx^{n} = \left[ {r\left( {s - s_{0} } \right) - f\left( x \right)} \right]$$

then the $\Delta R/R_{0}$ as a function of strain *x* can be described by Eq. (17),

17 $$\frac{\Delta R}{{R_{0} }} = \left( {1 + Ax} \right){\text{ exp}}\left( {wx^{n} } \right) - 1,$$

where *A*, *w* and *n* are constants.

For Eq. (14), during the actual experiment, as strain increases, TPU/CB sensors with excessive stretch deformation makes all conducting paths broken ($N \to 0$), leading to the conductive of TPU/CB sensor out of operation. It can be calculated limit as follows:

18a $$f\left( x \right) = \ln \frac{N}{{N_{0} }}$$

18b $$\mathop {\lim }\limits_{N \to 0} f\left( x \right) = \mathop {\lim }\limits_{N \to 0} \ln \frac{N}{{N_{0} }} = - \infty$$

then substituting $\left[ {f\left( x \right) \to - \infty } \right]{ }$ into Eq. (16),

19 $$wx^{n} = \left[ {r\left( {s - s_{0} } \right) - f\left( x \right)} \right] \approx - f\left( x \right)$$

20 $$\frac{N}{{N_{0} }} = {\text{exp}}\left( { - wx^{n} } \right).$$

**Fig. 8:**
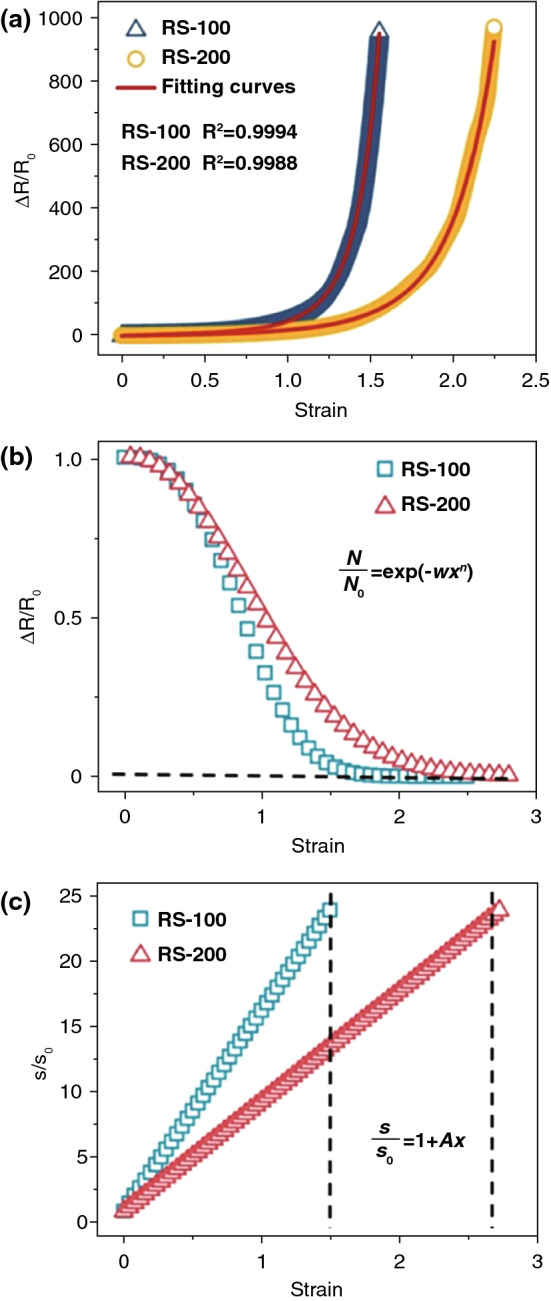
**a** Experimental and theoretical data of ${\Delta }R/R_{0}$ as a function of strain. Variation of **b** conductive pathways and **c** adjacent conductive particles of RS-100 and RS-200 TPU/CB strain sensor

Figure 8a shows the experimental curves of the strain-dependent $\Delta R/R_{0}$ fitted by model Eq. (17). The three parameters (*A*, *w*, *n*) calculated from the results of the tests as well as *R*^*2*^ are listed in Table 1. It is shown that our model Eq. (17) can indeed be used to describe and be in good consistent with the electrical resistance of all TPU/CB strain sensors. Based on Eqs. (13) and (20) as well as parameters in Table 1, the changes of conductive paths $\frac{N}{{N_{0} }}$ and adjacent conductive particles distance $\frac{s}{{s_{0} }}$ are plotted in Fig. 8b, c. As shown in Fig. 8b, the conductive paths of RS-100 and RS-200 samples begin to approach to 0 when the strain is approximately 1.5 and 2.2, respectively, which corresponding with the strain values of improving $\Delta R/R_{0}$ rapid in Fig. 8a. In Fig. 8c, it is apparent that the adjacent conductive particles distance of RS-100 growth faster more than that of RS-200 samples. In addition, when $\frac{s}{{s_{0} }}$ are almost 23, the strain is 1.5 for RS-100 sample and 2.6 for RS-200 sample. The strain values are corresponding with the strains dependent $\frac{N}{{N_{0} }} = 0$.

**Table 1:** The calculated parameters of Eq. (17) model

	*A*	*w*	*n*	*R*^*2*^
100	15.30 ± 0.26	1.050 ± 0.012	2.826 ± 0.017	0.9994
200	8.40 ± 0.24	0.716 ± 0.015	2.076 ± 0.018	0.9988

As shown in Fig. 8 and Table 1, it can be deduced clearly that for TPU/CB strain sensors with diverse rotating speed, the variation of conductive paths and adjacent conductive particles distance is different due to the different scaffold network structures under the same deformation, result in the variant conductive sensitivity. According to the mathematical model Eq. (17), the three parameters (*A*, *w*, *n*) reflect the changing level of the distance between conducting particles and the number of conductive paths directly during the deformation of the samples. For RS-100 and RS-200 samples, the three parameters are smaller at higher speeds (shown in Table 1). Parameter *A* is on behalf of the change speed of distance of adjacent CB particles. Comparing with RS-200 samples, the *A* of RS-100 samples is bigger, which indicate that the distance of conductive particles in RS-100 samples increase faster during the stretching process. Parameters *w* and *n* represent the variation of the number of conductive paths, which plays a vital role in electrical conductivity and sensitivity. This indicates that RS-100 samples with higher parameters *w* and *n* show more remarkable sensitivity than RS-200 samples. Based on parameter calculated from Eq. (15), Eqs. (13) and (20) were provided to accurately predict the variation distance of adjacent conductive particles and the number of conductive paths among the stretching process, respectively. Generally speaking, it is difficult to measure the distance of adjacent conductive particles and conductive paths. These models make it possible to quantitative predict the change of conductive paths and adjacent conductive particles.

### Sensing Application of TPU/CB Strain Sensor in Human Motions

**Fig. 9:**
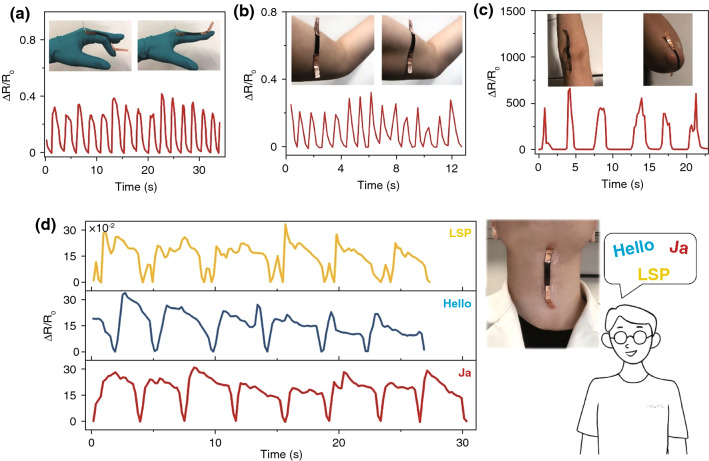
Sensing application of the TPU/CB strain sensor in human motions. **a** Finger bending, **b** muscle tremor, **c** elbow bending, **d** speaking different words with sample fixing at neck, inset: schematic illustrations of finger bending, muscle tremor, elbow bending and speaking

The TPU/CB strain sensors prepared in this paper are possible to monitor human motions due to excellent durability and flexibility performance, wide response range and high sensitivity. As shown in Fig. 9a, b, strain sensors were fixed on a finger and arm of the volunteer, the series of small response signals can be acquired instantaneously when volunteer motioned. The large human motion response was tested by bending elbow with more than 100% strain as well (Fig. 9c). Moreover, TPU/CB strain sensor could appear different response signals from various forms motions. For example, the response signals of extending/bending finger and muscle tremor have a significant difference. In Fig. 9d, a TPU/CB strain sensor was attached at neck and successfully captured response of slight movement of the volunteer speaking three words with different syllables, “Ja,” “Hello” and “LSP.” Given the excellent pressure-sensitive properties of TPU/CB strain sensor, corresponding response signs of three words were all detected exactly. In addition, it can be observed obviously difference among the three curves due to the vibration of different vocal cord, which will make different slight deformation of sensor. Otherwise, the number of speaking words and speaking frequency can be calculated through analyzing the response signals.

## Conclusion

In summary, a high-performance strain sensor was fabricated through embedding CB particles into the TPU fibrous film based on electrospinning and ultrasonication methods with high sensitive, wide stretchable strain range, low-cost and facile industrial production. The morphologies and electromechanical properties of RS-100 and RS-200 samples were studied and compared, which revealed the effect of rotating speed of electrospinning collection device on the stereoscopic scaffold network structures and fiber diameters of TPU/CB composites. Through adjusting rotating speed of collection device in electrospinning process, the fiber diameters and interweave-overlap get changed under the stress field, resulting in the scaffold structure of TPU matrix and conductive paths built by CB particles changed, which presents different conductive resistance under stress stimuli in the macroscopic. The TPU/CB strain sensors displayed prominent electromechanical performance with very wide workable stretching range, ultrahigh sensitivity, fast response time, and good stability and reproducibility, as well as economic availability and processing convenience, which present high competitive for intelligent terminals application in practical production. Because of its ultrahigh sensitivity, this sensor can be used for monitoring subtle or large human motions. Such as finger bending, muscle tremor and vocal cord vibration. A mathematic model was provided to describe the response performance of TPU/CB strain sensors, the changes of internal conductive particles and conductive paths were revealed as well. Last but not least, this article provides a novel strategy that rotating speed of collection device in electrospinning process plays a major roll of the fibrous film structure forming, determining the electromechanical of strain sensors, which fills a gap in fabricating high-sensitive, wearable, flexible strain sensors through electrospinning technology.

## Supplementary Information

Below is the link to the supplementary information.

Supplementary material 1 (PDF 519 kb)
