# Supplementary material for: Highly Sensitive Ultrathin Flexible Thermoplastic Polyurethane/Carbon Black Fibrous Film Strain Sensor with Adjustable Scaffold Networks
Source: Nanomicro Lett. 2021 Jan 25;13:64. doi: 10.1007/s40820-021-00592-9 (PMC8187661; doi:10.1007/s40820-021-00592-9)
Supplement: Supplementary file 1 — Supplementary material 1 (PDF 519 kb) [file 40820_2021_592_MOESM1_ESM.pdf]

Supporting Information for

Highly Sensitive Ultrathin Flexible Thermoplastic Polyurethane/Carbon Black Fibrous Film Strain Sensor with Adjustable Scaffold Networks

Xin Wang[1, 2], Xianhu Liu[1, 2, *], Dirk W. Schubert[1, 3, *]

[1]Institute of Polymer Materials, Friedrich-Alexander-University Erlangen-Nuremberg, Martensstr. 7, 91058, Erlangen, Germany

[2]National Engineering Research Center for Advanced Polymer Processing Technology, Zhengzhou University, Zhengzhou 450002, P. R. China

[3]Bavarian Polymer Institute, Dr. Mack-Strasse 77, 90762, Fürth, Germany

*Corresponding authors. E-mail: xianhu.liu@zzu.edu.cn (Xianhu Liu), dirk.schubert@fau.de (Dirk W. Schubert)

Supplementary Figures

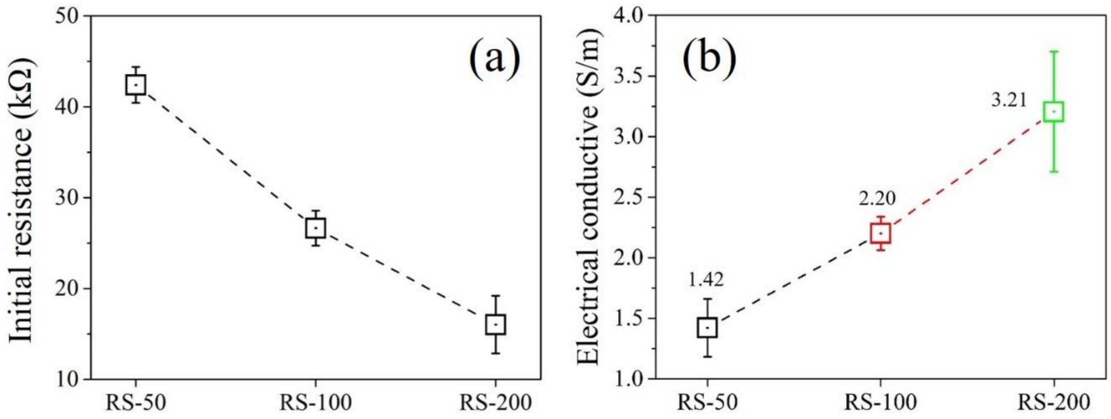

Fig. S1 a Initial resistance R_0 and **b** Electrical conductivity of TPU/CB strain sensors with different rotating speed

For the electrical conductivity measurement of TPU/CB strain sensors, both ends of the rectangular shape samples were attached copper tape as electrode. The initial resistance R_0 was measured and electrical conductivity σ was then calculated as:

$$\sigma = \frac{L}{R \times S_m} \quad \text{(S1)}$$

where R is the measured electrical resistance of sample, L is the distance between both coated ends, S_m is the area of cross sectional of strain sensor [S1]. As shown in Fig. S1, for TPU/CB strain sensor, the initial resistance decreased while the electrical

conductive increases with the rotating speed increasing. The initial resistance of RS-50 is almost 43 kΩ, RS-100 is 26 kΩ and RS-200 is 16 kΩ respectively. The electrical conductive of RS-50 is 1.42 S/m, which is not beneficial for fabricating wearable sensor [S2].

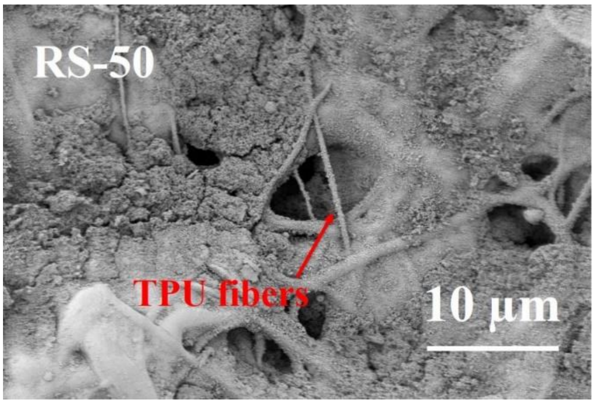

Fig. S2 SEM images of TPU/CB strain sensor with 50 rpm

In electrospinning processing, TPU fibers were spun to film on collection device with applied rotating speed. While it is against to eliminate the solvent completely of TPU mixture under low level applied rotating speed. The microstructures of TPU/CB strain sensor with 50 rpm were showed in Fig. S2. Compared with Figure 1c&e in, it is hard to observe the specific scaffold structure in Fig. S2.

As Figs. S1 and S2 showed above, in this work, 100 rpm and 200 rpm were chosen as the applied rotation speed of collection device for the fabrication of TPU fibrous film.

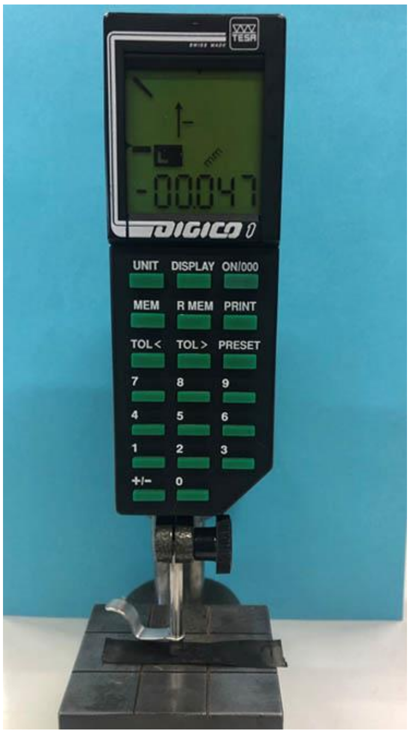

Fig. S3 The thickness of TPU/CB strain sensor is about 50 μm

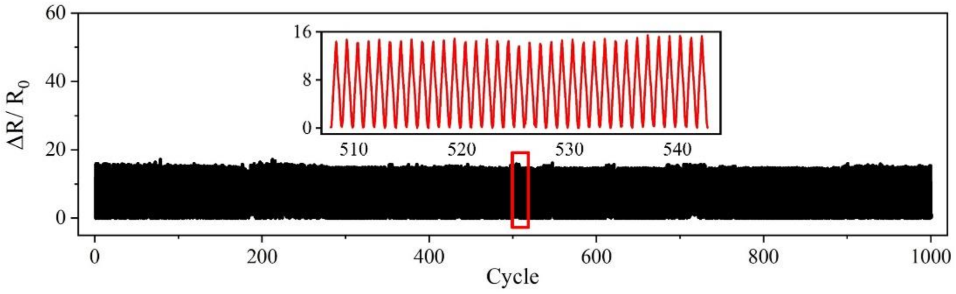

Fig. S4 1000 stretching-releasing cycles toward the strain variation from 0 to 50%

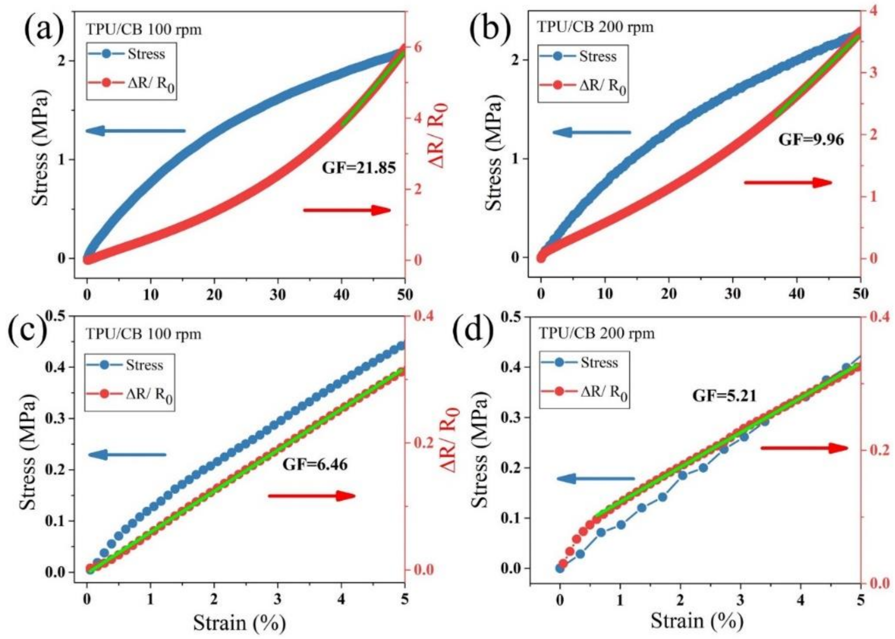

Fig. S5 The normalized change of **a** RS-100 and **b** RS-200 under 0-50% strain and under **c, d** 0-5% in tensile stress and electrical resistance ($\Delta R/R_0$) vs. strain.

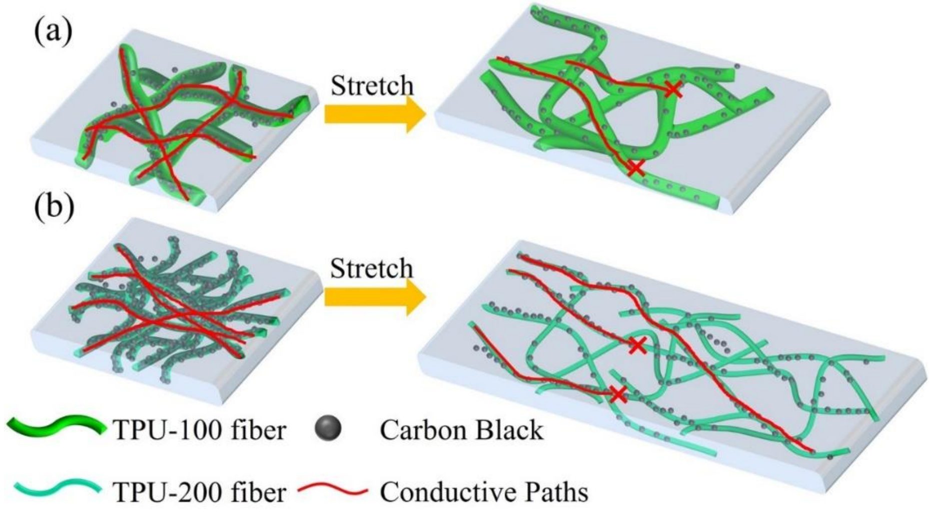

Fig. S6 Schematics of the morphology change of stretched (c) RS-100 and (d) RS-200 strain sensor

Supplementary References

[S1] H.G. Xu, M.C. Qu, D.W. Schubert, Conductivity of poly(methyl methacrylate) composite films filled with ultra-high aspect ratio carbon fibers. Compos. Sci. Technol. **181**, 107690 (2019). https://doi.org/10.1016/j.compscitech.2019.107690

[S2] Y. Wang, Y. Ji, Y. Zhou, Y. Wang, G. Zheng et al., Ultra-stretchable, sensitive and durable strain sensors based on polydopamine encapsulated carbon nanotubes/ elastic bands. J. Mater. Chem. C **6**, 8160 (2018). https://doi.org/0.1039/c8tc02702a
